# Heterogeneity of Primary Ciliary Dyskinesia Gene Variants: A Genetic Database Analysis in Russia

**DOI:** 10.3390/ijms262311674

**Published:** 2025-12-02

**Authors:** Elena I. Kondratyeva, Sergey N. Avdeev, Tatiana A. Kyian, Oksana P. Ryzhkova, Yuliya L. Melyanovskaya, Victoria V. Zabnenkova, Maria V. Bulakh, Zamira M. Merzhoeva, Artem V. Bukhonin, Natalia V. Trushenko, Baina B. Lavginova, Daria O. Zhukova, Sergey I. Kutsev

**Affiliations:** 1 Research Centre for Medical Genetics, 1 Moskvorechye St., Moscow 115522, Russia; 2 Pulmonology Department, Sechenov First Moscow State Medical University (Sechenov University), Healthcare Ministry of Russia, Trubetskaya St. 8, Build. 2, Moscow 119991, Russia

**Keywords:** primary ciliary dyskinesia, diagnostics, molecular genetic testing, sequencing, database

## Abstract

Primary ciliary dyskinesia (PCD) is a rare hereditary disorder belonging to the group of ciliopathies, with autosomal recessive, autosomal dominant, and, less frequently, X-linked inheritance patterns. The aim of this study was to investigate the genetic heterogeneity of the Russian population of PCD patients based on national registry data. The study included patients with PCD confirmed by molecular genetic testing. Quantitative data were analyzed using non-parametric statistical methods. Differences were considered statistically significant at *p* < 0.05. The study included 109 patients with PCD. Molecular genetic testing identified pathogenic variants in 29 autosomal recessive genes. The analysis of pathogenic variant distribution in the Russian PCD cohort revealed the highest number of changes in the *DNAH5* and *DNAH11* genes. 26 genetic variants in 13 genes were identified for the first time in the Russian population. Variants in the *DNAH5* gene were significantly more frequent in Kartagener’s syndrome (KS) patients (32/55%) compared to those without KS (11/21.5%) (χ^2^ = 12.8; *p* = 0.0004; OR = 4.48). Preliminary data indicate that the frequency spectrum of *DNAH5* and *DNAH11* genes in Russian patients is similar to international trends. Additionally, there is an accumulation of pathogenic variants in the *DNAH5*, *DNAH11*, *CCDC39,* and *CFAP300* genes.

## 1. Introduction

PCD is a rare inherited disease that belongs to the group of ciliopathies, with autosomal recessive, autosomal dominant, and, less frequently, X-linked inheritance patterns [1]. More than 60 genes associated with PCD have been identified. According to a study from 34 PCD centers in Europe, Asia and South America, 46 genes and 908 genetic variants associated with PCD were identified in 1236 patients. The study confirmed 687 (56%) homozygous variants, 528 (43%) compound heterozygous variants, and 20 (2%) hemizygous variants. X-linked variants were reported in the *OFD1*, *DNAAF6*, and *RPGR* genes, along with 1 (0.1%) autosomal dominant variant in the *FOXJ1* gene [2].

It is well established that the disease is caused by a defect in the ultrastructure of motile cilia in the respiratory tract epithelium and other similar structures (e.g., flagella of sperm cells, fallopian tube villi, ventricular ependyma). These defects in ciliary assembly and function are caused by specific genes in which variants have been identified [3].

The inability of the cilia to move synchronously leads to impaired mucociliary clearance and to the progression of respiratory tract disease, recurrent otitis media, hearing loss, chronic rhinosinusitis and impaired fertility. The initial symptoms manifest from the first days of life and continuously progress, ultimately leading to respiratory failure [4,5].

The diagnosis of PCD involves a set of investigation: measurement of nasal nitric oxide (nNO), high-speed video microscopy analysis (HSVM) to assess ciliary beat frequency and pattern in viable ciliated cells (ex vivo or in vitro using Air-Liquid Interface (ALI) cultures); immunofluorescent staining of various structural proteins, transmission electron microscopy (TEM) and genetic testing to identify pathogenic variants in genes associated with PCD. According to the European Respiratory Society (ERS) guidelines, the diagnosis of PCD is established using three methods: HSVM, TEM and molecular genetic testing [6]. The American Thoracic Society (ATS) also recommends the use of TEM and genetic diagnostics [7,8]. The recommendations of the ERS experts were used in the Russian registry.

In recent years, more than 60 genes responsible for PCD have been described in the scientific record. Significant genetic heterogeneity is associated primarily with five key genes: *DNAH5*, *DNAH11*, *DNAI1*, *CCDC39* and *CCDC40*. These genes represent the most frequently mutated PCD-associated genes in the global population. By analyzing genetic databases, Hannah et al. demonstrated that the frequency of pathogenic variants in the five principal PCD genes exhibits significant variability across different ethnicities and geographic regions. Mutations in these genes account for a significant proportion of the global genetic basis of PCD and have therefore served as the foundation for developing targeted therapies [9].

The main research objective was to investigate the genetic heterogeneity of the Russian patient population with PCD based on the 2024 registry data.

## 2. Results

In 2024, the Research Centre for Medical Genetics database enrolled 350 patients. Molecular genetic testing was performed for 188 patients (53.7%), among whom 148 (78%) were found to carry monoallelic or biallelic pathogenic variants. A definitive genetic diagnosis of PCD was established in 109 patients (73.6%), while the remaining 39 patients (26.4%) required functional assays for variant confirmation. A total of 121 genetic variants (95.9%) were found in 29 genes with autosomal recessive inheritance. Variants in genes with autosomal dominant (n = 2 (1.8%) FOXJ1 gene) and X-linked recessive (n = 3 (2.7%) OFD1 gene) modes of inheritance were much less common.

**Figure 1:**
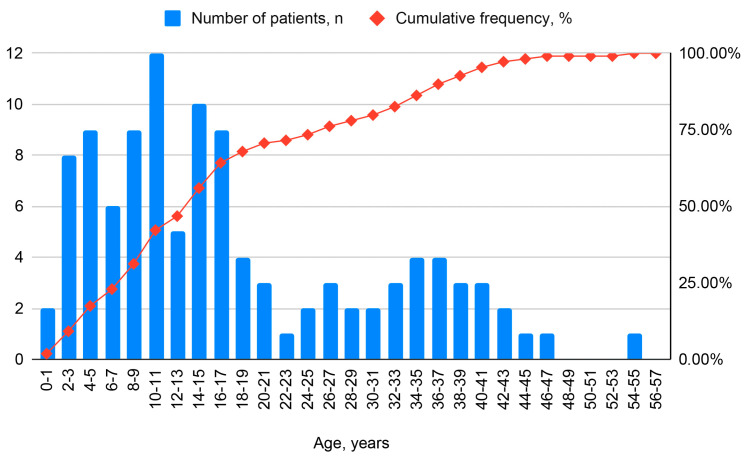
Histogram of the patient age.

The database included 109 patients with PCD. In the current year, 88 (80.7%) patients were followed up, while 21 (19.3%) were lost to follow-up. The mean age of patients in 2024 was 17.7 ± 13.1 years, with a median age of 14 (IQR 8–27) years. The oldest patient in 2024 was 55 years old (located in Moscow), and the youngest was 0.1 years old. Adult patients (≥18 years) constituted 34.9% of the cohort (Figure 1).

**Figure 2:**
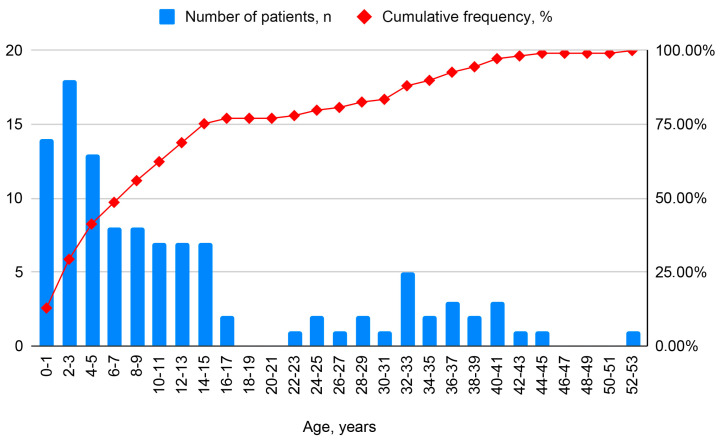
Histogram of age at diagnosis in different patient groups.

The study population showed a female predominance (55%) over male patients (45%). The mean age at diagnosis was 14.6 ± 13.8 years, with a Me of 10 years (IQR 8–25.8) (Table 1). The median age at diagnosis was 7 years (IQR 2.3–10.0) in pediatric patients and 32 years (IQR 17–38.5) in adults. For patients with Kartagener’s syndrome (KS), the age at diagnosis was 11.6 years (IQR 0.6–14) (Figure 2).

Genetic testing was performed for all 109 patients (100%) in the cohort. The analysis identified 121 unique pathogenic variants across 29 different genes in this Russian PCD patient cohort. The majority, 116 variants (95.9%), were found in genes with autosomal recessive inheritance. Variants in autosomal dominant genes were found in 2 patients (1.8%), specifically in the *FOXJ1* gene, and variants in X-linked recessive genes were found in 3 patients (2.7%), specifically in the *OFD1* gene; these occurred significantly less frequently.

Among patients with identified variants, homozygous genotypes were present in 39 patients (35.8%), compound heterozygous in 65 (59.6%), hemizygous in 3 (2.7%), and heterozygous in 2 (1.8%). Variants in autosomal recessive genes were collectively identified in 208 alleles, compared to 2 alleles for the autosomal dominant variant and 3 alleles for the X-linked recessive variants.

Analysis of the pathogenic variant distribution within the Russian PCD cohort showed that the highest number of changes was detected in the *DNAH5* and *DNAH11* genes. The frequency of identified genetic variants in descending order is presented in Table 2.

When the patients were stratified into groups, 58 (53%) were classified as having KS and 51 (47%) without it. Analysis revealed that variants in the *DNAH5* gene were significantly more frequent in the KS group, affecting 32 patients (55%), compared to 11 patients (21.5%) in the non-KS group (χ^2^ = 12.8; *p* = 0.0004; OR = 4.48; 5% CI 0.92–10.4) (Table 3).

The genes: *DNAAF3*, *LRRC50/DNAAF1*, *CCDC103*, *CCDC40*, *CFAP52*, *DNAH6*, *DNAH7*, *RSPH4A*, *CCDC164/DRC1*, *DNAH17*, *FSIP2*, *GAS8/DRC4*, *SPAG1*, *RSPH9*, and *CEP164*—each identified in single patients and therefore not included in the table.

Twenty-six novel genetic variants across 13 genes: *DNAH5*, *OFD1*, *DNAH14*, *DNAH2*, *DNAH11*, *DNAAF11/LRRC6*, *DNAAF4*, *DNAAF1*, *CFAP221*, *CCDC39*, *DNAH6*, *CFAP300*, and *CEP164* were identified for the first time in the Russian population compared to global data (Table 4).

By the type of mutation, the identified variants were: missense—45 variants (37.2%), nonsense—30 variants (24.8%), frameshift mutation—27 (22.3%), splice site mutation—15 (12.4%); copy number variation—1 (0.8%), deletions/duplications without frameshift—2 (1.8%), intronic variant—1 (0.8%) (Table S1). In a patient with a pathogenic nucleotide sequence variant in exon 3 of the CFAP300 gene (chr11:102058881 CT>C) in a homo/hemizygous state, resulting in a frameshift mutation (NM_032930.3: c.200delT, p.(Phe67fs)), a likely pathogenic variant was identified in the PKD1 gene in a hemizygous state (consistent with autosomal dominant inheritance). No significant variants were found in the remaining patients.

## 3. Discussion

A comparative analysis of key patient characteristics and the age at diagnosis across various international PCD specialist centers is of significant interest. According to a multicenter study encompassing data from 34 global PCD centers, the median age of the study population was 21.6 years (IQR 15.4–32.2 years). The cohort comprised 428 individuals (35%) under 18 years of age and 808 (65%) aged 18 years or older. Data on the age at diagnosis were available for 947 individuals, revealing a median age at diagnosis of 10 years (IQR 4.4–17 years). Notably, the median age at diagnosis for participants with laterality defects was 8 years (IQR 1.08–16.3 years), compared to 11 years (IQR 6–17.9 years) for those without such defects (*p* < 0.0001) [2]. In the Russian cohort, the mean age at diagnosis was 14.6 ± 13.8 years, with a Me of 10 years (IQR 8–25.8) (Table 1). The median age at diagnosis was 7 years (IQR 2.3–10.0) in pediatric patients and 32 years (IQR 17–38.5) in adults. For patients with Kartagener syndrome, the median age at diagnosis was 11.6 years (IQR 0.6–14).

In a cohort of 1236 patients with PCD, 908 pathogenic variants across 46 PCD-associated genes were identified. The genotypic distribution revealed 687 homozygous (56%) and 528 compound heterozygous (43%) variants. Hemizygous, X-linked variants (in *OFD1*, *DNAAF6*, and *RPGR*) were diagnosed in 20 individuals (2%), while a single patient presented with an autosomal dominant variant in the *FOXJ1* gene [2].

In the Russian cohort, the autosomal recessive inheritance pattern with compound heterozygous variants predominated (95.9%). In contrast, data from 34 centers worldwide show a predominance of homozygous variants, comprising 56% of cases. Regarding autosomal dominant forms, a variant in the *FOXJ1* gene was identified in a single patient (0.08%) within the multinational cohort of 1236 patients. In comparison, this variant was found in two patients (1.8%) within the Russian cohort of 109 patients [2].

According to the Russian genetic database, the frequency of variants in the *DNAH5* gene aligned with the multicenter international study, ranking first in both cohorts (33.9% and 22%, respectively). The *DNAH11* gene ranked second in both populations (9.2% and 11%). Statistically significant differences were observed between the Russian cohort and global data for variants in the *DNAH5* and *CFAP300* genes. It is important to note that these findings are preliminary and may be subject to change with a larger sample size. The observed disparities are likely attributable to recurrent variants that have become prevalent due to genetic drift (founder effect).

Beyond this point, the frequency spectrum of other genes diverged. In the Russian cohort, variants in the *CCDC39* and *CFAP300* genes shared the third rank (6.4% each), whereas in the international dataset, the *CCDC40* gene occupied the third position (9%) (Table 5) [2].

The genes *LRRC50/DNAAF1*, *DNAH7*, *DNAAF3*, *CCDC164/DRC1*, *RSPH4A*, *CEP164*, *CFAP52*, *DNAH6*, *DNAH17*, *FSIP2*, *CCDC103*, *GAS8/DRC4*, *SPAG1*, and *RSPH9* were each identified in single patients and are consequently not presented in the table.

In a Chinese study involving 244 patients with PCD, of whom 116 (47.5%) were female, 120 (49.2%) were diagnosed with KS, and 189 (77.5%) were diagnosed with PCD before 18 years of age. Among these patients, 120 (49.2%) showed first symptoms during the neonatal period, 85 (34.8%) in early childhood, and 3 (1.2%) in adulthood. For the 237 patients with precisely documented age at diagnosis, the mean age was 13.1 years.

Among 142 genetically characterized patients, pathogenic variants were identified in 25 PCD-associated genes. The genotypic distribution was as follows: 105 patients (73.9%) had compound heterozygous mutations, 27 (19.0%) had homozygous mutations, 6 (4.2%) had X-linked recessive mutations, and 4 (2.8%) had no detectable mutations. Of all identified variants, over half were loss-of-function mutations, comprising frameshift (26.7%), nonsense (21.0%), splicing (9.9%), and deletion (2.9%) variants. The most frequently identified genes were *DNAH5* (21.1%), *DNAH11* (18.3%), *CCDC39* (9.2%), *CCDC40* (6.3%), *HYDIN* (4.9%), *CCNO* (4.9%), and *DNAAF3* (4.9%) (Table 6) [11].

The genetic profile of the Russian cohort shares similarities with Chinese data, particularly concerning the high-frequency genes *DNAH5*, *DNAH11*, and *CCDC39*, though *HYDIN* variants appear more common in the Chinese population [12].

In the Chinese cohort, over half of all identified variants were loss-of-function mutations, comprising frameshift (26.7%), nonsense (21.0%), splicing (9.9%), and deletion (2.9%) variants. The Russian cohort exhibited a different mutational spectrum. The most prevalent variant type was missense (37.2%), followed by nonsense (24.8%), frameshift (22.3%), splicing site mutations (12.4%), copy number variations (0.8%), in-frame deletions/duplications (1.8%), and intronic variants (0.8%).

Recent years have witnessed significant genetic heterogeneity in the five key PCD-associated genes (*DNAH5*, *DNAH11*, *DNAI1*, *CCDC39*, and *CCDC40*) across western populations [9,13].

A study conducted by Hannah WB et al. investigated the global prevalence and ethnic heterogeneity of variants in major PCD-associated genes, including *DNAI1*. Analysis of genetic variant databases revealed that *DNAI1* mutations are relatively frequent in the PCD patient population. The specific frequency of *DNAI1* variations demonstrates population-specific differences, being identified in approximately 10–15% of patients in western countries [9].

## 4. Materials and Methods

### 4.1. Clinical Methods

a. Patient history: a comprehensive anamnestic assessment.

b. PCD Risk Stratification: For patients presenting with a persistent wet cough and clinical suspicion of PCD, the PICADAR predictive tool was employed in accordance with international guidelines. A threshold score of >5 points was applied, corresponding to a reported sensitivity of 0.90 and specificity of 0.75 [14].

### 4.2. Molecular Genetic Methods

Patient DNA analysis was performed on the Illumina NextSeqDx 500 next-generation sequencer (Illumina, Inc. San Diego, CA, USA) using SeqCap EZ HyperCap Workflow reagents (Roche, Basel, Switzerland). Detected variants were named according to HGVS nomenclature [15]. Whole-exome sequencing data were processed using a standard automated data analysis algorithm provided by Illumina and available at https://basespace.illumina.com [16]. Population frequencies of identified variants were assessed using reference samples from the 1000 Genomes Project (https://www.internationalgenome.org), ESP6500: Exome Sequencing Project v. 6500, gnomAD (v.3.1.2), and RuExac (a database of variants obtained through whole-exome sequencing (WES, CES) at the Research Centre for Medical Genetics) [17]. Pathogenicity assessment of this variant in the studied samples was conducted in accordance with Russian guidelines for interpreting data obtained by massive parallel sequencing (MPS) [18,19].

### 4.3. Statistical Analyses

Statistical analyses were performed using IBM SPSS Statistics software, version 26 (IBM Co., Armonk, NY, USA). The distribution of the quantitative variables was non-normal and the data are presented as median and interquartile range (IQR), reported as Me (IQR). Categorical variables are summarized as absolute numbers and percentages (%). Statistical analysis of quantitative data was performed using the Mann–Whitney U test. Categorical data were analyzed using Pearson’s Chi-squared test or Fisher’s exact test. The Wilcoxon signed-rank test was used for comparison of paired samples (pre-post analysis). A *p*-value of ≤0.05 was deemed statistically significant.

## 5. Conclusions

Our findings confirm the critical importance of molecular genetic testing for the timely and accurate determination of the genetic basis of PCD. Preliminary data demonstrate that the frequency distribution of variants in the *DNAH5* and *DNAH11* genes in the Russian population aligns with international trends. Therefore, the optimal diagnostic approach is the use of whole-exome (or whole-genome) sequencing. Furthermore, we observed significant clustering of pathogenic variants in the *DNAH5*, *DNAH11*, *CCDC39*, and *CFAP300* genes. The development of targeted genetic panels for PCD diagnosis represents a promising future direction. These diagnostic advancements will be crucial for improving patient quality of life, particularly with the advent of targeted therapies.

## Figures and Tables

**Table 1:** The mean age and median age at diagnosis.

Parameter	Child (<18 Years)	Adult (≥18 Years)	All
Total number n (%)	71 (65.1)	38 (34.9)	109 (100)
Age at diagnosis, years			
M ± SD	6.6 ± 4.8	27.9 ± 13.8	14.6 ± 13.8
Me (IQR)	7 (2.3–10.0)	32 (17–38.5)	10 (8–25.8)

**Table 2:** Number of genes, genetic variants and their frequency in the Russian population.

№	Gene	Number of Identified Unique Genetic Variants	Inheritance Pattern	Number of Patients with Pathogenic Variants, n	Number of Patients with Pathogenic Variants, %
1	*DNAH5*	43	AR	43	39.4
2	*DNAH11*	13	AR	10	9.2
3	*CCDC39*	5	AR	7	6.4
4	*C11ORF70/CFAP300*	4	AR	7	6.4
5	*LRRC6/DNAAF11*	3	AR	5	4.6
6	*OFD1*	3	XLR	3	2.7
7	*HYDIN*	5	AR	3	2.7
8	*DNAH9*	4	AR	3	2.7
9	*CCDC40*	2	AR	2	1.8
10	*CFAP221*	1	AR	2	1.8
11	*DNAH14*	4	AR	2	1.8
12	*CCDC114/ODAD1*	4	AR	2	1.8
13	*DNAL1*	1	AR	2	1.8
14	*DYX1C1/DNAAF4*	3	AR	2	1.8
15	*FOXJ1*	2	AD	2	1.8
16	*LRRC50/DNAAF1*	2	AR	1	0.9
17	*DNAH7*	2	AR	1	0.9
18	*DNAAF3*	2	AR	1	0.9
19	*CCDC164/DRC1*	2	AR	1	0.9
20	*RSPH4A*	2	AR	1	0.9
21	*CEP164*	2	AR	1	0.9
22	*CFAP52*	2	AR	1	0.9
23	*DNAH6*	2	AR	1	0.9
24	*DNAH17*	2	AR	1	0.9
25	*FSIP2*	2	AR	1	0.9
26	*CCDC103*	1	AR	1	0.9
27	*GAS8/DRC4*	1	AR	1	0.9
28	*SPAG1*	1	AR	1	0.9
29	*RSPH9*	1	AR	1	0.9
	All genes	123		109	100%

Note: frequently occurring variants in genes according to the Varsome database [10].

**Table 3:** Frequency of PCD-associated genes in patients with and without Kartagener syndrome (KS).

№	Gene	Inheritance Pattern	KS, n	KS, %	Absence of KS, n	Absence of KS, %	*p*-Value
1	*DNAH5*	AR	32	55.0	11	21.5	0.0004
2	*DNAH11*	AR	5	8.6	5	9.8	0.748
3	*CCDC39*	AR	3	5.2	4	7.8	1
4	*DNAH9*	AR	0	0	3	5.8	-
5	*C11ORF70/CFAP300*	AR	5	8.6	2	4.0	0.4
6	*LRRC6/DNAAF11*	AR	1	1.7	4	7.8	0.2
7	*DNAH14*	AR	0	0	2	4.0	-
8	*HYDIN*	AR	0	0	3	5.8	-
9	*CCDC114/ODAD1*	AR	1	1.7	1	1.9	1
10	*OFD1*	XLR	0	0	3	5.8	-
11	*DYX1C1/DNAAF4*	AR	0	0	2	4.0	-
12	*CFAP221*	AR	0	0	2	4.0	-
13	*DNAL1*	AR	2	3.5	0	0.00	-
14	*RSPH4A*	AR	1	1.7	0	0.00	-
15	*FOXJ1*	AD	1	1.7	1	1.9	1

**Table 4:** New onset genetic variants in PCD-associated genes identified in the Russian population.

№	Gene	Unreported Genetic Variant Previously	gnomAD v3.1.2 Number of Homozygotes	gnomAD v3.1.2 Allele Frequency	ACMG	P
1	*DNAH5*	c.12850dup, p.(Tyr4284LeufsTer14)	n/d	n/d	PVS1, PM2, PM3	P
		c.3074dupC, p.(Ala1026fs)	n/d	n/d	PVS1, PM2, PM3	P
		c.8390T>G, p.(Leu2797Arg)	n/d	n/d	PM2, PM3	VoUS
		c.6813C>A, p.(Cys2271Ter)	n/d	n/d	PVS1, PM2	LP
		c.12216del, p.(Tyr4072Ter)	n/d	n/d	PVS1, PM2	LP
		c.13604_13609del, p.(Val4535_Tyr4536del)	n/d	n/d	PM2, PM3	VoUS
2	*DNAH11*	c.13387_13444dup, p.(Arg4482LysfsTer20)	n/d	n/d	PVS1, PM2	LP
		c.5461-3T>G, p.(?)	n/d	n/d	PM2, PM3	VoUS
		c.8572G>A, p.(Gly2858Ser)	0	0.0002037	PM2	VoUS
		c.8363A>G, p.(His2788Arg)	n/d	n/d	PM2	VoUS
3	*OFD1*	c.2674C>T, p.(Gln892Ter)	n/d	n/d	PVS1, PM2	LP
4	*DNAH14*	c.9011G>C, p.(Arg3004Pro)	n/d	n/d	PM2	VoUS
		c.12068C>T, p.(Pro4023Leu)	n/d	n/d	PM2	VoUS
5	*DNAH2*	c.5372C>T, p.Thr1791Met	0	0.00003942	PM2	VoUS
6	*DNAAF11/LRRC6*	c.574C>G, p.(Gln192Glu)	0	0.001197	PM2	VoUS
		c.1011A>G, p.(Gln337Gln)	0	0.00003942	PM2	VoUS
7	*DNAAF4*	c.430dup, p.(Ile144AsnfsTer8)	0	0.00001994	PVS1, PM2, PM3	P
8	*DNAAF1*	c.1384C>T, p.(Gln462Ter)	n/d	n/d	PVS1, PM2	LP
		c.655T>C, p.(Cys219Arg)	0	0.00006580	PM2, PM3	P
9	*CFAP221*	c.1641dup, p.(Asn548GlnfsTer6)	0	0.0001646	PVS1, PM2	LP
10	*CCDC39*	c.2492_2496del, p.Met831ThrfsTer7	n/d	n/d	PVS1, PM2, PM3	P
11	*DNAH6*	c.11669G>A, p.(Arg3890His)	0	0.0005784	PM2	VoUS
		c.11612-42A>G, p.?	0	0.0008870	PM2, PP3	VoUS
12	*CFAP300*	c.289G>T, p.(Glu97Ter)	0	0.000006576	PVS1, PM2	LP
13	*CEP164*	c. 1865G>A, p.Arg622Gln	0	0.0001446	PM2, PP3	VoUS
		c.3055C>T, p.Gln1019Ter	0	0.00003944	PVS1, PM2	LP

ACMG criteria: P—pathogenic, LP—likely pathogenic, VoUS—uncertain significance.

**Table 5:** Comparative analysis of PCD-associated gene frequency in the Russian Federation and the International Study (2024).

Gene	Russia (N = 109)	Patient, %	International Study (N = 1236)	Patient, % (International Study)	Place (International Study)	*p*
*DNAH5 (603335)*	43	39.4	275	22.0	1	0.009
*DNAH11 (603339)*	10	9.2	142	11.0	2	0.5
*CCDC39 (613798)*	7	6.4	66	5.0	5	0.7
*C11ORF70/CFAP300 (618058)*	7	6.4	22	1.8	13	0.006
*LRRC6*/*DNAAF11 (614930)*	5	4.6	44	3.6	8	0.3
*OFD1 (300170)*	3	2.7	3	0.24	18	0.009
*HYDIN (610812)*	3	2.7	42	3.4	9	0.8
*DNAH9 (603330)*	3	5.5	5	0.4	16	0.00008
*CCDC40 (613799)*	2	1.8	115	9.0	3	0.004
*CFAP221*	2	1.8	1	0.08	22	0.02
*DNAH14*	2	1.8	0	–	-	–
*CCDC114/ODAD1 (615038)*	2	1.8	37	3.0	10	0.5
*DNAL1 (610062)*	2	1.8	0	–	–	–
*DYX1C1/DNAAF4 (608706)*	2	1.8	35	2.8	11	0.4
*FOXJ1 (602291)*	2	1.8	1	0.1	21	0.02

**Table 6:** Comparative analysis of genetic heterogeneity in PCD patients from the Russian and Chinese cohort.

Russian Cohort		Chinese Cohort	
Gene	%	Gene	%
*DNAH5*	39.4	*DNAH5*	21.1
*DNAH11*	9.2	*DNAH11*	18.3
*CCDC39*	6.4	*CCDC39*	9.2
*C11ORF70/CFAP300*	6.4	*CCDC40*	6.3
*LRRC6/DNAAF11*	4.6	*HYDIN*	4.9
*OFD1*	2.7	*CCNO*	4.9
*HYDIN*	2.7	*DNAAF3*	4.9
*DNAH9*	5.5	*DNAH1*	3.5
*CCDC40*	2	*DNAAF11 (LRRC6)*	3.5

## Data Availability

The original contributions presented in this study are included in the article and Supplementary Materials. Further inquiries can be directed to the corresponding author.

## Supplementary Materials

The following supporting information can be downloaded at: https://www.mdpi.com/article/10.3390/ijms262311674/s1.
