# Supplementary material for: Heterogeneity of Primary Ciliary Dyskinesia Gene Variants: A Genetic Database Analysis in Russia
Source: Int J Mol Sci. 2025 Dec 2;26(23):11674. doi: 10.3390/ijms262311674 (PMC12692196; doi:10.3390/ijms262311674)
Supplement: Supplementary file 1 [file ijms-26-11674-s001.zip › ijms-4008327-supplementary.pdf]

Table S1. Characteristics of nucleotide sequence variants in genes responsible for PCD in the Russian Federation in 2024.

№	Inheritance pattern	Inheritance pattern	cDNA	p.	Type of variant	gnomAD v3.1.2 Number of Homozygotes	gnomAD v3.1.2 Allele Frequency
1	*DNAH5 (603335)*	AR	c.3599-2A>G	-	site splicing mutation	n/d	n/d
2	*DNAH5 (603335)*	AR	c.12779C>T	p.(Asp4260Val)	missense	n/d	n/d
3	*DNAH5 (603335)*	AR	c.10700T>C	p.(Leu3567Ser)	missense	0	0.00003942
4	*DNAH5 (603335)*	AR	c.12216del	p.(Tyr4072Ter)	nonsense	n/d	n/d
5	*DNAH5 (603335)*	AR	c.8390T>G	p.(Leu2797Arg)	missense	n/d	n/d
6	*DNAH5 (603335)*	AR	c.11653C>T	p.(Arg3885*)	nonsense	0	0.00001973
7	*DNAH5 (603335)*	AR	c.13604_13609del	p.(Val4535_Tyr4536del)	deletion without frameshift	n/d	n/d
8	*DNAH5 (603335)*	AR	c.8403G>A	p.Trp2801*	nonsense	n/d	n/d
9	*DNAH5 (603335)*	AR	c.1354A>T	p.Lys452*	nonsense	n/d	n/d
10	*DNAH5 (603335)*	AR	c.7596T>A	p.Tyr2532Ter	nonsense	n/d	n/d
11	*DNAH5 (603335)*	AR	c.2224C>T	p.Arg742*	nonsense	0	0.00001315
12	*DNAH5 (603335)*	AR	c.3598G>A	p.Ala1200Thr	missense	n/d	n/d
13	*DNAH5 (603335)*	AR	c.5563dup	p.Ile1855AsnfsTer6	reading frameshift	0	0.00004601
14	*DNAH5 (603335)*	AR	c.5177T>C	p.Leu1726Pro	missense	0	0.00001314
15	*DNAH5 (603335)*	AR	c.1207C>T	p.Gln403*	nonsense	n/d	n/d
16	*DNAH5 (603335)*	AR	c.10420-2A>G	-	splice site mutation	n/d	n/d
17	*DNAH5 (603335)*	AR	c.8029C>T	p.(Arg2677*)	nonsense	0	0.000006579
18	*DNAH5 (603335)*	AR	c.8498G>A	p.(Arg2833His)	missense	0	0.000006571
19	*DNAH5 (603335)*	AR	c.8497C>T	p.(Arg2833Cys)	missense	0	0.00001315
20	*DNAH5 (603335)*	AR	c.2880_2881del	p.(His961PhefsTer31)	reading frameshift	n/d	n/d
21	*DNAH5 (603335)*	AR	c.6185_6195dupCTTT TATCTTT	p.Thr2066fs	reading frameshift	n/d	n/d
22	*DNAH5 (603335)*	AR	c.3034_3041delGCAA GCGT	p.Ala1012fs	reading frameshift	n/d	n/d
23	*DNAH5 (603335)*	AR	c.7508_7510dup	p.(Arg2503dup)	non-frameshift duplication	n/d	n/d
24	*DNAH5 (603335)*	AR	c.6763C>T	p.(Arg2255*)	nonsense	0	0.00002631
25	*DNAH5 (603335)*	AR	c.2734del	p.(Glu912LysfsTer14)	reading frameshift	n/d	n/d
26	*DNAH5 (603335)*	AR	c.12523G>A	p.(Val4175Met)	missense	0	0.0003482
27	*DNAH5 (603335)*	AR	c.1089+1G>A	-	splice site mutation	0	0.00005256
28	*DNAH5 (603335)*	AR	c.2052+3G>T	-	splice site mutation	n/d	n/d
29	*DNAH5 (603335)*	AR	c.10815del	p.(Pro3606HisfsTer23)	reading frameshift	0	0.0001906
30	*DNAH5 (603335)*	AR	c.12850dup	p.(Tyr4284LeufsTer14)	reading frameshift	n/d	n/d
31	*DNAH5 (603335)*	AR	c.232C>T	p.(Arg78Ter)	nonsense	0	0.000006577
32	*DNAH5 (603335)*	AR	c.8314C>T	p. Arg2772Ter	nonsense	0	0.000006580

33	*DNAH5 (603335)*	AR	c.4488G>A	p.(Trp1496Ter)	nonsense	n/d	n/d
34	*DNAH5 (603335)*	AR	c.13533_13534del	p.(Met4511IlefsTer5)	reading frameshift	n/d	n/d
35	*DNAH5 (603335)*	AR	c.10616G>A	p.Arg3539His	missense	0	0.00005261
36	*DNAH5 (603335)*	AR	c.6444+2T>C	-	splice site mutation	n/d	n/d
37	*DNAH5 (603335)*	AR	c.10441C>T	p.Arg3481*	nonsense	0	0.000006575
38	*DNAH5 (603335)*	AR	c.3074dupC	p.(Ala1026fs)	reading frameshift	n/d	n/d
39	*DNAH5 (603335)*	AR	c.6305G>A	p.(Arg2102His)	missense	0	0.000006573
40	*DNAH5 (603335)*	AR	c.8440_8447del	p.(Glu2814*)	reading frameshift	0	0.00001314
41	*DNAH5 (603335)*	AR	c.13369T>C	p.(Trp4457Arg)	missense	n/d	n/d
42	*DNAH5 (603335)*	AR	c.9124C>T	p.(Arg3042*)	nonsense	n/d	n/d
43	*DNAH5 (603335)*	AR	c.6813C>A	p.(Cys2271Ter)	nonsense	n/d	n/d
44	*DNAH11 (603339)*	AR	c.6664C>T	p.(Arg2222Ter)	nonsense	0	0.000006575
45	*DNAH11 (603339)*	AR	c.5593C>A	p.(Arg1865Arg)	missense	n/d	n/d
46	*DNAH11 (603339)*	AR	c.2966G>A	p.(Arg989Gln)	missense	0	0.00001315
47	*DNAH11 (603339)*	AR	c.7294C>T	p.(Arg2432Trp)	missense	2	0.0004736
48	*DNAH11 (603339)*	AR	c.4360_4361delinsTT	p.(Glu1454Leu)	missense	n/d	n/d
49	*DNAH11 (603339)*	AR	c.8501T>C	p.(Met2834Thr)	missense	0	0.000006570
50	*DNAH11 (603339)*	AR	c.13140del	p.(Phe4380LeufsTer7)	reading frameshift	0	0.000006572
51	*DNAH11 (603339)*	AR	c.10751A>C	p.(His3584Pro)	missense	n/d	n/d
52	*DNAH11 (603339)*	AR	c.8363A>G	p.(His2788Arg)	missense	n/d	n/d
53	*DNAH11 (603339)*	AR	c.2417del	p.Gln806ArgfsTer11	reading frameshift	n/d	n/d
54	*DNAH11 (603339)*	AR	c.2432del	p.Gly811AlafsTer6	reading frameshift	n/d	n/d
55	*DNAH11 (603339)*	AR	c.1231G>T	p.Gly411*	missense	n/d	n/d
56	*DNAH11 (603339)*	AR	c.13373C>T	p.Leu4019fs	reading frameshift	n/d	n/d
57	*CCDC39 (613798)*	AR	c.2492_2496del	p.(Met831Thrfs*7)	reading frameshift	n/d	n/d
58	*CCDC39 (613798)*	AR	c.811del	p.(Ser271ValfsTer21)	reading frameshift	0	0.000004344
59	*CCDC39 (613798)*	AR	c.610-2A>G	-	splice site mutation	0	0.0001117
60	*CCDC39 (613798)*	AR	c.2098C>T	p.(Gln700Ter)	nonsense	n/d	n/d
61	*CCDC39 (613798)*	AR	c.2497_2498del	p.(Gln833Valfs*6)	reading frameshift	0	0.00001533
62	*HYDIN (610812)*	AR	c.950G>A	p.Arg317Gln	missense	0	0.000007067
63	*HYDIN (610812)*	AR	c.11695G>A	p.Val3899Met	missense	0	0.00001314
64	*HYDIN (610812)*	AR	c.1443T>G	p.(Tyr481Ter)	nonsense	0	0.000006570
65	*HYDIN (610812)*	AR	c.1974+1G>A	-	splice site mutation	n/d	n/d
66	*HYDIN (610812)*	AR	c.8791C>T	p.(Arg2931Cys)	missense	n/d	n/d
67	*DNAH9 (603330)*	AR	c.1571G>A	p.(Arg524Gln)	missense	0	0.0001710
68	*DNAH9 (603330)*	AR	c.1604A>G	p.(Asp535Gly)	missense	0	0.0001709
69	*DNAH9 (603330)*	AR	c.7176_7177delinsC	p.(Asp2393ThrfsTer11)	reading frameshift	n/d	n/d

70	*DNAH9 (603330)*	AR	c.10648C>T	p.(Arg3550Trp)	missense	0	0.00002630
71	*DNAH14*	AR	c.9685C>T	p.(Arg3229Ter)	nonsense	n/d	n/d
72	*DNAH14*	AR	c.13639-2del	-	splice site mutation	n/d	n/d
73	*DNAH14*	AR	c.5977+3_5977+6delAAGT	-	splice site mutation	n/d	n/d
74	*DNAH14*	AR	c.10765del	p.(Thr3589Glnfs*28)	reading frameshift	n/d	n/d
75	*C11ORF70/CFAP300 (618058)*	AR	c.198_200delinsCC	p.(Phe67Profs*10)	reading frameshift	n/d	n/d
76	*C11ORF70/CFAP300 (618058)*	AR	c.289G>T	p.(Glu97Ter)	nonsense	0	0.000006576
77	*C11ORF70/CFAP300 (618058)*	AR	c.195_197delTTTinsCC	p.(Phe661LeufsTer11)	reading frameshift	n/d	n/d
78	*C11ORF70/CFAP300 (618058)*	AR	c.200delT	p.(Phe67fs)	reading frameshift	n/d	n/d
79	*CCDC114 (615038) / ODAD1*	AR	c.1502+5G>A	-	splice site mutation	0	0.00008542
80	*CCDC114 (615038) / ODAD1*	AR	c.413del	p.(Pro138ArgfsTer18)	reading frameshift	n/d	n/d
81	*CCDC114 (615038) / ODAD1*	AR	c.281_300del	p.(Asp94GlyfsTer33)	reading frameshift	n/d	n/d
82	*CCDC114 (615038) / ODAD1*	AR	chr19:48122933-48334195	-	copy number variations	n/d	n/d
83	*LRRC6 (614930)/DNAAF11*	AR	c.436G>C	p.(Asp146His)	missense	0	0.00009868
84	*LRRC6 (614930)/DNAAF11*	AR	c.1011A>G	p.(Gln337Gln)	synonymic variants	0	0.00003942
85	*LRRC6 (614930)/DNAAF11*	AR	c.79_80del	p.(Ser27ValfsTer13)	reading frameshift	0	0.0001117
86	*DYX1C1/DNAAF4 (608706)*	AR	c.988C>T	p.(Arg330Trp)	missense	0	0.00002631
87	*DYX1C1/DNAAF4 (608706)*	AR	c.583del	p.(Ile195Ter)	nonsense	0	0.00008579
88	*DYX1C1/DNAAF4 (608706)*	AR	c.430dup	p.(Ile144AsnfsTer8)	reading frameshift	0	0.00001994
89	*OFD1 (300170)*	XLR	c.2674C>T	p.(Gln892Ter)	nonsense	n/d	n/d
90	*OFD1 (300170)*	XLR	c.2635G>T	p.(Glu879*)	nonsense	n/d	n/d
91	*OFD1 (300170)*	XLR	c.2725C>T	p.(Arg909Ter)	nonsense	n/d	n/d
92	*LRRC50/DNAAF1 (613190)*	AR	c.655T>C	p.(Cys219Arg)	missense	0	0.00006580
93	*LRRC50/DNAAF1 (613190)*	AR	c.1384C>T	p.(Gln462Ter)	nonsense	n/d	n/d
94	*DNAH7*	AR	c.9292-15T>G	-	splice site mutation	n/d	n/d
95	*DNAH7*	AR	c.2576C>T	p.(Ala859Val)	missense	n/d	n/d
96	*DNAAF3 (614566)*	AR	c.1324C>T	p.(Gln442Ter)	nonsense	n/d	n/d
97	*DNAAF3 (614566)*	AR	c.1238+5G>C	-	splice site mutation	n/d	n/d
98	*CCDC164/DRC1 (615288)*	AR	c.352C>T	p.(Gln118*)	nonsense	0	0.0002828

99	*CCDC164/DRC1 (615288)*	AR	c.1345C>T	p.(Gln449*)	nonsense	n/d	n/d
100	*RSPH4A (612647)*	AR	c.347_348del	p.(Val116AspfsTer3)	reading frameshift	n/d	n/d
101	*RSPH4A (612647)*	AR	c.1690del	p.(Gln564LysfsTer20)	reading frameshift	n/d	n/d
102	*CCDC40 (613799)*	AR	c.2740dup	p.(Ile914AsnfsTer84)	reading frameshift	0	0.000006569
103	*CCDC40 (613799)*	AR	c.2440C>T	p.(Arg814Ter)	nonsense	0	0.00001314
104	*CEP164*	AR	c.3055C>T	p.Gln1019Ter	nonsense	0	0.00003944
105	*CEP164*	AR	c. 1865G>A	p.(Arg622Gln)	missense	0	0.0001446
106	*CFAP52*	AR	c.636+3A>T	-	splice site mutation	n/d	n/d
107	*CFAP52*	AR	c.1026-2A>G	-	splice site mutation	n/d	n/d
108	*DNAH6*	AR	c.11669G>A	p.(Arg3890His)	missense	0	0.0005784
109	*DNAH6*	AR	c.11612-42A>G	-	intronic variant	0	0.0008870
110	*DNAH17*	AR	c.12583_12585del	p.(Glu4195del)	Deletion without frameshift	0	0.000006580
111	*DNAH17*	AR	c.10202C>T	p.(Pro3401Leu)	missense	0	0.0006113
112	*FSIP2*	AR	c.12793G>T	p.(Gly4265Term)	nonsense	n/d	n/d
113	*FSIP2*	AR	c.16671A>C	p.(Glu5557Asp)	missense	n/d	n/d
114	*FOXJ1 (602291)*	AD	c.15G>A	p.(Trp5Ter)	nonsense	n/d	n/d
115	*FOXJ1 (602291)*	AD	chr17:g.76132156_76144966del	-	copy number variations	n/d	n/d
116	*DNAL1 (610062)*	AR	c.23_24del	p.(Lys8Argfs*16)	reading frameshift	0	0.00001997
117	*CCDC103 (614677)*	AR	c.461A>C	p.(His154Pro)	missense	0	0.001248
118	*GAS8/DRC4 (605178)*	AR	NC_000016.10:g.(?*90019732)*(90043244_?)del	-	copy number variations	n/d	n/d
119	*SPAG1 (603395)*	AR	c.2014C>T	p.(Gln672Ter)	nonsense	0	0.00009200
120	*RSPH9 (612648)*	AR	c.488_498del	p.(Pro163HisfsTer8)	reading frameshift	n/d	n/d
121	*CFAP221*	AR	c.1641dup	p.(Asn548GlnfsTer6)	reading frameshift	0	0.0001646
